# Crania Britannica

**Published:** 1854-04

**Authors:** John M. Galt


ART. II.— *Crania Britannica.* A new Ethnological Work.

We give place with pleasure to the following announcement of a forthcoming work, furnished us by Dr. John M. Galt, of the Eastern Lunatic Asylum of Virginia.—ED.

We have lately received from our friends the authors, the prospectus of an important work relative to Ethnology. The following is the title which they propose to give to this important undertaking:

*Crania Britannica, or Delineations of the Skulls of the Aboriginal Inhabitants of the British Islands, and of the Races immediately succeeding them; together with notices of their other remains.* By JOSEPH BARNARD DAVIS, F. S. A., Member of the Royal College of Surgeons of England, of the Archæological Institute of Great Britain, Associate of the Ethnological Society, and of the British Archæological Association; and JOHN THURNAW, M. D., F. S. A., Licentiate of the Royal College of Physicians of London, Member of the Archæological Institute of Great Britain and Ireland, of the British Archæological Association, and of the Ethnological Society of London, author of Papers on the Ethnology and Archæology of England.

Here we have an example of the energy and activity of science; as in geology, she traces out the history of the earth in the animal remains which characterize each stratum of the mighty mass. For here the hand of science again brings to our notice, and elicits truths, from the dust of those who have ended life's dark journey during ages that are past. And in both of these subjects we have something worthy of our consideration. In the microcosm, the great globe which pursues without rest, or a moment's cessation, its eternal course through the fathomless abysses of space; and in man the microcosm, "few and evil" as are the days of his pilgrimage—yet still made after "the image of God," the "marvel of marvels" as he is called by Plato, "distinguished link in Being's endless chain," as he is described by Young.

But to return to more homely language: from the eminent qualifications of the authors—from their zeal in the undertaking—from their talent and the opportunities for information which they possess to so great a degree, we are satisfied that this work will constitute a valuable addition to the knowledge which we at present have of ethnology. And we can vouch for their sparing no pains or expense toward rendering this production an honor, alike to its authors and to the age in which they live; we would mention further that it will be dedicated, by permission, to the Queen of England, and that it has already obtained the sanction of numerous learned and scientific gentlemen, both in Great Britain and in other countries.

The cost of the work, which will come out in six decades, may be estimated at about thirty dollars, or one guinea for each decade, published quarterly—to be paid for on delivery. It is to be "privately printed," issued in London, and strictly confined to subscribers, whose names are requested to be communicated to the authors only, that its further progress may be insured; for it is distinctly stated by them that it is proposed to publish the series of decades "if a sufficient number of subscribers can be obtained to prevent a pecuniary loss." Each of the six decades is intended to contain ten lithographic plates, in Imperial quarto, with descriptive letter press, giving the history of the exhumation of each cranium, an account of the antiquities disinterred with it, accompanied by figures, &c., together with exact admeasurements. The lithographs will, in every instance where it is possible, be carefully drawn on the stone from the skulls themselves, of the full and exact size of nature; and so as not merely to preserve the correct outline, but to render the surface and actual relief of the cranium.

Mr. Davis has lately written me word that subscriptions, the names of subscribers, &c., may be transmitted either to himself, directed Joseph Barnard Davis, Esq., Shelton, Staffordshire, England, or to Franklin Peale, Esq., United States Mint, Philadelphia, Pennsylvania.

JOHN M. GALT.

January 30, 1854.

